# Fibrocalcific phenotypes in calcific aortic valve disease: imaging characterization, treatment timing, and efficacy assessment

**DOI:** 10.3389/fcvm.2026.1997440

**Published:** 2026-10-27

**Authors:** Feng Liu, Zhaohong Zeng, Zhian Qian, Wentong Li, Yuting Huang, Ziyou Liu

**Affiliations:** 1 First Clinical Medical College, Gannan Medical University, Ganzhou, China; 2 Key Laboratory of Prevention and Treatment of Cardiovascular and Cerebrovascular Diseases of Ministry of Education, First Affiliated Hospital of Gannan Medical University, Gannan Medical University, Ganzhou, China; 3 Cardiology Medical Center, First Affiliated Hospital of Gannan Medical University, Gannan Medical University, Ganzhou, China

**Keywords:** aortic stenosis, calcific aortic valve disease, calcification, computed tomography angiography, efficacy endpoints, sex differences, treatment timing, valvular fibrosis

## Abstract

Calcific aortic valve disease involves two structural processes, fibrous matrix remodeling and mineral deposition, that contribute unequally between patients, so leaflets of similar hemodynamic severity need not share the same tissue composition. Histologic and multi-omics studies have mapped this heterogeneity across sex, age, and valve morphology, and contrast-enhanced computed tomography angiography (CTA) has brought compositional assessment into living patients and into longitudinal follow-up. Calcific burden behaves consistently: it tracks stenosis severity, predicts progression, and carries prognostic weight. Findings for noncalcified tissue are less settled and depend on the population studied and on how the measurement is made. Whether total fibrocalcific volume performs well as a structural endpoint and whether CTA-derived noncalcified tissue volume adds predictive value beyond calcification are consequently two separate questions, and the literature often merges them. Pharmacologic trials in patients have almost always measured calcification progression or hemodynamics rather than longitudinal change in the fibrous matrix. Randomized lipid-lowering and mineral-targeted trials in patients have been negative, whereas animal and cell experiments show that established mineral lesions, cell states, and function can sometimes be modified, although these responses do not necessarily move together. This review integrates histology, multimodality imaging, clinical cohorts, pharmacologic trials, and preclinical intervention studies to define the fibrocalcific phenotype, distinguish prevention from slowing and reversal, and align therapeutic timing with endpoints at the levels of activity, tissue burden, valve function, myocardial response, and clinical events. Longitudinal measurement of leaflet composition and validation of treatment-induced change against functional outcomes remain the central gaps.

## 1 Introduction

Calcific aortic valve disease (CAVD) spans a continuum that runs from leaflet sclerosis, thickening, and focal calcification through to hemodynamically significant stenosis, and its prevalence climbs steeply with age. Among the 5,201 participants aged 65 years or older in the Cardiovascular Health Study, aortic sclerosis was present in 26% and aortic stenosis in 2%. The Tromsø population study put the prevalence of aortic stenosis at 3.9% in people aged 70–79 years and 9.8% in those aged 80–89 years, a pronounced age gradient (1, 2).

Population aging has pushed the aggregate burden upward. Using Global Burden of Disease 2021 data, one analysis estimated that the number of people living with nonrheumatic CAVD grew from approximately 4.98 million in 1992 to 13.32 million in 2021, an increase of about 168%, while the age-standardized prevalence rose from 130.82 to 158.35 per 100,000. Because prevalence rose after standardization for age, demographic change alone does not account for the trend (3).

The structural substrate is not mineral alone. Collagen accumulates, matrix organization changes, and leaflets thicken; fibrosis and calcification both stiffen the leaflet and restrict its opening, but their relative contributions differ from patient to patient. On average, human histologic studies have found lower calcific burden and higher proportions of collagen and dense connective tissue in women at comparable hemodynamic severity. The distributions overlap substantially, however, and sex does not determine an individual patient's tissue composition. Equivalent degrees of stenosis can thus rest on different tissue compositions, and calcific burden alone does not capture the structural heterogeneity of leaflet disease (4).

Surgical and transcatheter aortic valve replacement (TAVR) have transformed the management of severe aortic stenosis, yet no pharmacologic therapy modifies the native-valve disease process. The 2025 ESC/EACTS guidelines state that no medical treatment has been shown to alter the natural history of aortic stenosis. Between the first leaflet abnormalities and functional obstruction, the disease may advance silently for years. A growing patient population and the absence of any disease-modifying option have made attenuation of structural damage and functional deterioration a central target of drug development (5).

Imaging has begun to bring tissue heterogeneity into *in vivo* assessment. Noncontrast computed tomography (CT) quantifies accumulated mineral, whereas contrast-enhanced computed tomography angiography (CTA) can estimate calcified, noncalcified, and total fibrocalcific tissue volumes. Longitudinal work has linked change in total fibrocalcific volume to progression of the transvalvular pressure gradient, extending structural assessment beyond the calcium score. Noncalcified volume, mineral burden, and hemodynamic status each describe a different aspect of leaflet disease and its functional consequences (6).

Pharmacologic studies in patients have concentrated on progression of calcification and hemodynamics; data that track the leaflet fibrous matrix directly remain scarce. Reduced mineralization activity, a fall in mineral burden, matrix remodeling, and improved valve function are responses at different levels, and their directions and time courses need not coincide. The relation between tissue state at the moment treatment begins, the biological process a drug targets, and the endpoint chosen for follow-up runs through studies of prevention, slowing, and reversal alike. This review organizes that problem around the fibrocalcific phenotype. We ask what histology and imaging can currently say about lesion composition, how composition relates to severity, progression, and outcome, and what would count as evidence that a treatment-induced tissue response yields durable functional benefit. The aim is not to catalogue every candidate drug but to clarify which questions each class of evidence can and cannot answer.

### 1.1 Literature search and evidence synthesis

This review is narrative rather than systematic. PubMed was searched on August 7, 2026, and Europe PMC on August 9, 2026; supplementary searches on human drug exposure and multimodality imaging were updated to September 12, 2026. The PubMed search combined Medical Subject Headings and title/abstract terms for calcific aortic valve disease, aortic stenosis, and aortic sclerosis with pharmacologic, dietary, and molecular intervention terms, without language, species, or publication-type filters. Ten Europe PMC queries addressed histology, imaging, mechanisms, epidemiology, genetics, recent fibrocalcific phenotypes (limited to records from January 1, 2024), positron emission tomography, prognosis, myocardial fibrosis, and antifibrotic interventions, excluding reviews, editorials, and comments; for each query, the 100 most-cited records were retrieved. Citation ranking favors established work; reference tracking and targeted retrieval of studies identified by title or identifier supplemented the database results. The archived query strings are available from the corresponding authors on request.

Studies were selected purposively for their relevance to tissue composition, imaging characterization, treatment timing, and efficacy assessment rather than against prospective eligibility criteria. Human histologic comparisons, imaging-validation studies, longitudinal cohorts, and randomized trials were prioritized, and experimental studies were included when treatment initiation and endpoints could be related to lesion stage. Guidelines and consensus documents provided clinical and measurement context. Screening was not performed in duplicate, and no formal risk-of-bias instrument was applied.

Evidence was synthesized qualitatively and by question, without pooling. Histology–imaging studies were compared by reference standard, component definition, agreement, and repeatability. For efficacy, randomized between-group estimates were weighted above nonrandomized comparisons, medication–histology associations, and experimental findings, and primary endpoints were distinguished from secondary, subgroup, and exploratory analyses. Mineralization activity, accumulated mineral, noncalcified tissue, hemodynamics, myocardial response, and clinical events were treated as separate outcome domains. Neither within-group change nor a terminal between-group difference was taken as evidence of reduction from the pretreatment baseline, and serial measurements in the same participants or animals were distinguished from comparisons with a separate baseline group. Apparent conflicts were examined against differences in population or model, baseline lesion state, exposure duration, measurement method, and follow-up, and against shared cohorts or analytic platforms. Information not reported in the source studies was not inferred.

## 2 Histologic basis and heterogeneity of fibrocalcific phenotypes

### 2.1 Leaflet remodeling and tissue composition

The normal aortic valve leaflet absorbs continuously changing pressure and deformation through a layered architecture. The collagen-rich fibrosa carries most of the tensile load, the elastin network on the ventricular side allows low-strain extension and recoil, and interlayer connections hold the structure together through repetitive loading. Layer-specific biaxial testing has shown that fibrosa and ventricularis respond differently in both nonlinearity and anisotropy, and the low-level tension generated by elastin restores collagen-fiber configuration after unloading. Composition and architecture matter equally to function (7, 8).

Early lesions typically appear beneath the endothelium of the aortic surface and in the adjacent fibrosa. Focal thickened areas in autopsy leaflets contain intracellular and extracellular lipid, microscopic mineral deposits, disrupted basement membrane, and infiltrating macrophages and T lymphocytes. Apolipoprotein B, apolipoprotein(a), and apolipoprotein E can be detected from morphologically early lesions through to late stenotic leaflets, which places lipoprotein retention and tissue injury across much of the disease course rather than at one end of it (9, 10).

Matrix remodeling is spatially uneven. In human calcified leaflets, the proteoglycans decorin, biglycan, and versican and the glycosaminoglycan hyaluronan concentrate around calcific nodules but are sparse within them, and perinodular composition differs between small prenodular deposits and mature nodules. The matrix immediately adjacent to a lesion is thus an active participant in cell adhesion and mechanical signaling and, at the same time, a local reservoir for lipid- and mineralization-related molecules (11).

Osteogenic molecules are expressed in calcified regions, and a subset of advanced valves develops organized ectopic bone. Human calcified aortic valves express osteopontin, bone sialoprotein, osteocalcin, and RUNX2-related markers; a separate study of surgical specimens identified mature lamellar bone, bone remodeling, and local neovascularization. Mineral deposition, an osteogenic cell state, and mature ectopic bone are three different things, and evidence for one is not evidence for another (12, 13).

Fibrotic and calcified regions of the same leaflet differ molecularly. In a multi-omics analysis of 25 surgical aortic valves, Schlotter and colleagues resolved distinct expression networks in macroscopically unaffected, fibrotic, and calcified regions, and cells derived from the fibrosa showed greater mineralization potential in culture. Even end-stage leaflets contain regions that differ in activity and composition, a point with practical consequences, since whole-valve averages will obscure local responses (14).

### 2.2 Cell states, matrix mechanics, and fibrosis–mineralization coupling

Valvular interstitial cells (VICs) maintain matrix homeostasis and drive pathologic collagen secretion, contraction, and mineralization. Single-cell maps of human aortic valves have identified multiple VIC, endothelial, and immune-cell subpopulations, along with enriched interstitial states and features consistent with endothelial-to-mesenchymal transition in diseased leaflets (15).

Myofibroblast-like and osteogenic states are not mutually exclusive. Work on sortilin identified a human VIC subset coexpressing inflammatory, myofibroblastic, and osteogenic genes, and sortilin-deficient mice subjected to mechanical aortic-valve injury developed less fibrosis, less calcification, and less stenosis. Multi-omic analysis of tissue-derived extracellular vesicles added another layer: valvular vesicles carry protein and microRNA networks capable of regulating VIC mineralization, including tissue-specific components related to Wnt signaling (16, 17).

Matrix mechanics and biochemical stimuli steer differentiation together. Yip et al. cultured porcine VICs on collagen gels and found that, under identical procalcific conditions, softer matrices favored mineralized aggregates containing viable cells with osteogenic expression, whereas stiffer matrices more readily produced myofibroblast contraction, apoptosis, and aggregate mineralization. The interaction between transforming growth factor beta 1 (TGF-*β*1) and *β*-catenin is similarly stiffness dependent, with fibrosa-like stiffness supporting stronger myofibroblast differentiation (18, 19).

What TGF-*β*1 does depends on the system in which it is studied. In ovine VIC cultures it induced cell aggregation, apoptosis, and subsequent nodule calcification. In three-dimensional cultures of intact leaflets it enhanced SMAD3 signaling and expression of alpha-smooth muscle actin (*α*SMA) and type I collagen while suppressing osteogenic signaling and hydroxyapatite deposition. The second system yielded a predominantly fibrotic response, a reminder that matrix deposition and mineral formation can move in opposite directions under the same stimulus (20, 21).

Sustained mechanical stimulation reaches the chromatin. In hydrogel studies, activated VICs on stiff matrices displayed open chromatin enriched for binding sites of mechanosensitive transcription factors and increased expression of contractile-fiber genes. Matrix stiffness is thereby linked to persistent cellular activation, which offers one mechanistic account of why drug responses differ between matrix environments (22).

Endothelial signaling restrains this feedback. Nitric oxide derived from valvular endothelium suppresses myofibroblast activation, osteogenic differentiation, and calcium deposition by VICs in three-dimensional culture, and endothelial nitric oxide synthase expression differs between the two leaflet surfaces. Genetic studies have tied NOTCH1 abnormalities to developmental valve morphology and to later calcification; inhibition of RUNX2 activity by NOTCH1 connects valve development with the regulation of mineralization in adult life (23, 24).

Lipid-related pathways cut across inflammation and mineralization. Variation at the LPA locus is associated with CT-detected aortic-valve calcification and with subsequent stenosis. Oxidized phospholipids and autotaxin carried on lipoprotein(a) [Lp(a)] can generate lysophosphatidic acid locally and activate inflammatory and osteogenic signaling. Circulating lipids, intraleaflet metabolism, and the matrix environment together set the intensity and persistence of these processes (25, 26).

Sphingolipid signaling provides a further link between lipid metabolism and osteogenic remodeling. Ceramide synthase 5 (CerS5) is a key enzyme in C16-ceramide synthesis. In wire-injured mice fed a Western diet, its deletion lowered transvalvular peak velocity, macrophage infiltration, and calcification without altering valve fibrosis, and these effects were absent on normal chow. CerS5 knockdown also reduced calcification of human VICs and inflammatory cytokine release from THP-1 cells (27). Sphingosine-1-phosphate (S1P) connects mechanical load with mineralization. Mechanical stretch increased sphingosine kinase 1–dependent S1P production in human VICs, and S1P receptor 2 (S1PR2) signaling promoted osteogenic differentiation through RUNX2/osteoprotegerin and GSK3*β*–Wnt–*β*-catenin pathways. Stenotic human valves exposed to higher wall shear stress contained more S1P and expressed more sphingosine kinase 1. In the wire-injury model, disease development was attenuated both by S1PR2 deficiency and by pharmacologic receptor inhibition started after injury (28).

### 2.3 Variation by sex, age, and valve morphology

Sex differences in calcific burden are reproducible but describe overlapping distributions, not separate groups. Among 665 patients with aortic stenosis, women carried a lower CT calcific burden at comparable echocardiographic severity, and the difference survived adjustment for body size. In 888 surgical patients, women had lower *ex vivo* valve weight, and valve weight correlated with the CT calcium score, tying the imaging measure to a whole-valve structural difference rather than to a scanning artifact (29, 30).

Collagen-level histology points the same way. Combining echocardiography, CT, and surgical tissue, Simard et al. found higher average fibrosis scores and greater proportions of collagen and dense connective tissue in women. Voisine et al. analyzed CT data from 434 patients alongside 138 surgical valves and also found higher collagen content in women, with strikingly low calcific burden in younger women with bicuspid valves. These are group-level differences, and their size varied with age and valve morphology (4, 31).

Differences in cellular regulation accompany these tissue differences. On hydrogels of physiologically relevant stiffness, female-derived VICs showed higher basal *α*SMA expression, and genes that partially escape X-chromosome inactivation contributed through Rho-associated protein kinase signaling. A separate study combining CTA with public transcriptomic datasets found a stronger fibrosis-related signature in women and enrichment of selected inflammatory and immune-cell signals in men (32, 33).

Spatial omics has begun to describe the regions surrounding lesions. A 2026 human single-cell and spatial-transcriptomic study reported more prominent AP-1–related expression in male VICs and enrichment of extracellular-matrix remodeling genes in women; SERPINE1 expression was higher in fibrous-dense regions of female lesions, and candidate macrophage–VIC interactions included SPP1–CD44 signaling. Tissue lipidomics has likewise shown sex-specific accumulation of sphingomyelins, ceramides, and related species (34, 35).

For an individual patient, tissue phenotype is what is measured, not what sex, age, or valve morphology would predict. These variables help interpret a measurement but cannot replace it, and sex is not an established criterion for selecting antifibrotic treatment.

## 3 From histology to imaging phenotypes

Because CTA and histology share terms but not measurements, the following definitions apply throughout. CTA calcified volume is the volume of leaflet voxels classified as mineralized tissue by the study-specific segmentation. CTA noncalcified tissue volume is the remaining segmented leaflet tissue, which includes collagen-rich matrix, other extracellular-matrix constituents, and cells. Total fibrocalcific volume is the sum of the two, and the noncalcified-to-calcified volume ratio expresses their relative amounts. Some CTA studies call the noncalcified compartment “fibrotic volume”; we report it as noncalcified tissue volume because it is not collagen specific. Fibrosis and collagen fraction keep their histologic or mechanistic meaning.

### 3.1 Histologic reference standards and validation of imaging quantification

Valve weight, collagen staining, and imaging-derived volume answer different questions. *Ex vivo* weight reflects total tissue mass; collagen area fraction gives the relative amount of one matrix component within the regions sampled; CTA estimates three-dimensional tissue volume in a living patient. Agreement between imaging and valve weight therefore supports assessment of overall burden, whereas the ability to resolve individual components has to be demonstrated against collagen, mineral, and other constituents directly.

Cartlidge et al. analyzed CTA in 164 patients with aortic stenosis and reported a correlation coefficient of 0.72 between total fibrocalcific volume and *ex vivo* valve weight, with noncalcified leaflet thickening examined in a pathologic subset. Grodecki et al. subsequently compared CTA composition with quantitative histology in 29 participants (19 with severe stenosis, 5 with moderate stenosis, and 5 controls), analyzing nine sections per valve. The CTA-derived noncalcified fraction correlated with the histologic fibrotic fraction (r = 0.92; intraclass correlation coefficient, 0.94), but the mean bias was −4.9 percentage points and the 95% limits of agreement spanned −18.5 to 8.7 percentage points. Strong group-level agreement thus coexisted with individual-level error wide enough to limit classification of single valves (36, 37).

The first study supports measurement of overall leaflet burden; the second, correspondence between CTA-defined fractions and sampled histology, although its agreement estimates rest on 29 mostly advanced valves. Correspondence is further constrained by two-dimensional section sampling, decalcification and fixation, and the three-dimensional distribution of lesions.

Table 1 summarizes representative histologic and imaging studies.

**Table 1:** Representative histologic and imaging studies of fibrocalcific phenotypes.

Study	Study design	Sample size	Disease stage	Principal endpoint	Principal findings
Simard 2017 (4)	Observational imaging–histology study with sex-matched comparison	125 surgical patients; 103 matched	Moderate-to-severe AS; tricuspid valves at surgical replacement	Sex differences in CT calcium burden and histologic fibrosis/collagen at comparable severity	At similar stenosis severity, women had less calcification and higher fibrosis/collagen measures on average.
Voisine 2020 (31)	Cross-sectional CT and surgical histology cohorts; sex-matched comparisons	434 CT participants; 138 surgical valves	AS of varying severity; bicuspid and tricuspid valves; histology at valve replacement	CT calcium density and histologic collagen and connective-tissue fractions by sex, age, and valve morphology	Average collagen content was higher in women; younger women with bicuspid valves could reach stenosis at a low calcific burden.
Cartlidge 2021 (36)	Post hoc cross-sectional analysis of two imaging cohorts; surgical validation subset	164 with AS; 38 controls; valve weight in 26	Mild-to-severe AS; nonstenotic controls	Association of CTA fibrocalcific volume with peak velocity and with explanted valve weight and histology	Total volume correlated with peak velocity and with valve weight (r = 0.72); in women it tracked severity better than calcium score alone.
Grodecki 2024 (37)	Prospective, two-center CTA–histology validation; secondary analysis	29 analyzed (34 enrolled)	19 severe AS; 5 moderate AS; 5 nonstenotic transplant controls	Agreement between CTA-defined noncalcified and calcified fractions and histologic tissue fractions	Close correlation of the CTA noncalcified fraction with the histologic fibrotic fraction (r = 0.92), but mean bias −4.9 percentage points with wide 95% limits of agreement (−18.5 to 8.7).
Lembo 2024 (6)	Post hoc imaging analysis of SALTIRE2 with longitudinal, external, and histologic validation	136 baseline; 90 at 1 year; 66 external; 41 surgical validation	Mild-to-severe AS; preserved LVEF	Reproducibility and validity of CTA fibrocalcific volume; association with severity and 1-year gradient progression	Total fibrocalcific volume rose by approximately 17% over 1 year and tracked gradient progression; baseline total volume predicted subsequent progression.
Lorenzatti 2025 (47)	Retrospective, single-center CTA study	651 patients	Symptomatic severe AS referred for TAVR planning	Annulus-indexed CTA tissue volumes and noncalcified-to-calcified volume ratio by sex and hemodynamic group	Higher ratio in women and in low-gradient disease, driven mainly by less calcified tissue; indexed noncalcified tissue volume did not differ significantly between groups.
Shu 2026 (48)	Retrospective, single-center cross-sectional CT–echocardiography study	448 patients	Mild-to-severe AS; normal flow and preserved LVEF	Association of tissue volume and density with peak velocity and indexed valve area, by sex and valve morphology	Calcification was consistently associated with severity; noncalcified-volume associations were weaker and, in women with bicuspid valves, depended on the grading criterion.

AS, aortic stenosis; CT, computed tomography; CTA, computed tomography angiography; LVEF, left ventricular ejection fraction; TAVR, transcatheter aortic valve replacement. CTA compartments labeled “fibrotic” in the original studies are reported here as noncalcified tissue; histologic fibrosis and collagen retain their tissue-based meaning. Total fibrocalcific volume is the sum of CTA calcified and noncalcified tissue volumes. Principal endpoint denotes the main outcome assessed and is not necessarily a prespecified primary endpoint. Cohort and validation-subset sample sizes are listed separately and are not additive. For Voisine et al., the CT cohort size (434) follows the full-text Methods, Results, and Figure 1; the abstract reports 411.

### 3.2 Diagnosis and longitudinal measurement of calcific burden

Noncontrast CT quantifies detectable mineral through the Agatston score or calcium volume, the former weighting lesion area by density and the latter counting voxels above a threshold. In an international multicenter study of 918 patients, optimal thresholds for identifying severe stenosis were approximately 1,377 AU in women and 2,062 AU in men, and both were associated with valve replacement or death. Among 485 patients with bicuspid valves, optimal thresholds varied by sex and ethnicity as well, with a higher threshold in Asian men. Sex-specific criteria rest on a comparatively mature evidence base, although the exact cut points remain population dependent (38, 39).

Calcific burden can also be recovered from contrast-enhanced CTA. A 2026 international multicenter study of 1,521 patients found that CTA calcium volume indexed to annular area correlated with peak velocity and with the noncontrast CT calcium score, and retained discriminatory performance for disease severity in an independent cohort. Existing CTA examinations can therefore be used for calcium quantification, with diagnostic and prognostic validity that belongs specifically to the mineral component (40).

Cross-sectional thresholds and longitudinal endpoints operate on different scales: one separates patients by severity, the other tracks change within a patient. Comparability of follow-up differences depends on consistency of acquisition, reconstruction, and segmentation, and mineral burdens near zero are especially sensitive to the detection threshold.

### 3.3 Quantification of total fibrocalcific and noncalcified tissue volumes

CTA quantification has moved from blood-pool–calibrated thresholds toward scan-specific distribution models. Cartlidge et al. calibrated tissue thresholds to blood-pool attenuation; Lembo et al. used a Gaussian mixture model to identify tissue-intensity distributions. Both approaches sum calcified and noncalcified volumes to yield total fibrocalcific volume, but they differ in segmentation rules, contrast enhancement, and boundary handling, which makes the noncalcified measurement method dependent.

In the Cartlidge cohort, total fibrocalcific volume correlated more strongly with peak velocity than did the Agatston score; in women the coefficients were 0.72 and 0.38. Lembo et al. extended the approach to 136 patients with an external validation cohort of 66. Total volume correlated with pressure gradient, and repeated scans gave a mean difference of −1% with limits of agreement from −4.5% to 2.8%. Over one year, total volume rose by approximately 17%, and its change correlated with change in mean gradient (*ρ* = 0.32); in women, baseline total volume was more strongly associated with subsequent gradient progression. The first study established cross-sectional association with severity, the second added repeatability and longitudinal tracking (6, 36).

Because total volume aggregates calcified and noncalcified components, its stronger associations may simply reflect the combined information of both. Repeatability, association with severity, and prediction of progression for the total measure say nothing about the incremental value of the noncalcified tissue component once calcification is accounted for. That question requires both components modeled jointly in the same cohort, a demonstrated improvement in model performance, and external validation. As matters stand, the evidence supports total fibrocalcific volume as a candidate structural endpoint far better than as a validated marker of response to antifibrotic therapy.

Absolute burden and relative composition carry different meanings. A higher noncalcified-to-calcified ratio may reflect more noncalcified tissue or less calcified tissue, and rising calcified volume lowers the ratio even when noncalcified volume is unchanged. The ratio is undefined at zero calcified volume and unstable near zero, where small segmentation errors in the denominator produce large ratio changes. We therefore recommend that future studies report, at a minimum, both absolute component volumes (mm^3^) and their sum, with the ratio given only where defined. Indexed volumes can be added if the indexing variable is stated. Prespecified handling of zero and near-zero denominators, with sensitivity analyses based on measurement precision, makes the influence of denominator error explicit.

### 3.4 Mineralization activity, tissue burden, and functional consequences

Positron emission tomography (PET) with 18F-sodium fluoride (18F-NaF) reports processes related to active mineralization through tracer binding to mineral surfaces. Uptake rises with the severity of aortic-valve disease across stages, and studies combining histology with longitudinal CT have connected tracer uptake to active calcification and to subsequent increases in calcium burden. Where CT records what has accumulated, PET reports what is currently happening (41, 42).

Uptake intensity, tissue-to-background ratio, and active calcification volume are computed differently and may diverge after treatment. Repeat-scan studies have shown that localization with contrast-enhanced CT, electrocardiographic gating, and motion correction improve the repeatability of selected measures, while acquisition timing and blood-pool correction remain critical for longitudinal comparison (43).

Echocardiography assesses obstruction through peak velocity, mean transvalvular gradient, and aortic valve area, each also influenced by transvalvular flow, left-ventricular function, and outflow-tract measurement. Cardiovascular magnetic resonance (CMR) contributes mainly information on myocardial remodeling under pressure overload. Across modalities the field can observe local activity, leaflet structure, hemodynamics, and ventricular consequences, and reading them together yields interpretations unavailable from any one of them (44, 45).

Table 2 compares the principal modalities and the questions each is equipped to answer.

**Table 2:** Principal measurement modalities and their roles in efficacy assessment.

Modality	Core measures and domain	Role in efficacy assessment	Principal sources of variation
Histology and chemical quantification	Collagen area fraction, leaflet thickness, mineral staining, tissue calcium content	Characterizes matrix remodeling, mineral deposition, and clearance; serves as the reference for imaging	Sampling site, decalcification, choice of area denominator, and divergence between stained area and total calcium
Noncontrast CT	Agatston score and calcium volume; accumulated mineral burden	Quantifies onset, progression, or regression of calcification	Acquisition and reconstruction parameters, segmentation threshold, and scan-rescan repeatability
Contrast-enhanced CTA	CTA-defined calcified and noncalcified tissue volumes, their sum (total fibrocalcific volume), and volume ratios	Quantifies leaflet tissue compartments and their longitudinal change	Blood-pool attenuation, partial-volume effects, calcium blooming, segmentation rules, and limited collagen specificity
18F-NaF PET	Uptake value, tissue-to-background ratio, active calcification volume; mineralization activity	Characterizes local disease activity and provides exploratory pharmacodynamic measures	Acquisition timing, blood-pool background, motion correction, and region-of-interest definition
Echocardiography	Peak velocity, mean transvalvular gradient, aortic valve area; valvular function	Assesses hemodynamic progression or improvement	Transvalvular flow, left-ventricular systolic function, outflow-tract measurement, and blood pressure
Cardiovascular magnetic resonance	Left-ventricular mass, extracellular volume, matrix volume, late gadolinium enhancement; downstream myocardial remodeling	Assesses ventricular response and change in risk	Measures myocardium rather than leaflet; extracellular-volume fraction reflects both cellular and matrix compartments

CMR, cardiovascular magnetic resonance; CT, computed tomography; CTA, computed tomography angiography; PET, positron emission tomography; ^18^F-NaF, ^18^F-sodium fluoride.

Representative sources: histology and CTA (6, 37); mineralization activity and hemodynamics (42, 44); myocardial remodeling (56).

## 4 Clinical associations of fibrocalcific phenotypes

### 4.1 Tissue composition and differences in disease severity

Discordance between aortic valve area and transvalvular-gradient grading has several causes. Among 646 patients with preserved left-ventricular ejection fraction, Clavel et al. found discordant grading in approximately 29%. CT calcific burden remained associated with pressure gradient after adjustment for flow, and systemic arterial compliance was independently associated with the gradient as well. Leaflet disease, flow state, and arterial load together shape the echocardiographic picture; a low gradient by itself says nothing definite about leaflet tissue composition (46).

Lorenzatti et al. compared relative composition with annulus-indexed volume directly in a pre-TAVR cohort of 651 patients. Women had lower indexed total fibrocalcific volume than men (230 vs. 293 mm^3^/cm^2^) and lower indexed calcified volume (85 vs. 149 mm^3^/cm^2^) but a higher noncalcified-to-calcified volume ratio (1.47 vs. 0.83); indexed noncalcified tissue volume did not differ significantly by sex (*P* = 0.805). Low-gradient patients likewise had a higher ratio without a significant difference in indexed noncalcified tissue volume. The higher ratio in women thus reflected less calcified tissue rather than more noncalcified tissue, and an individual's phenotype has to be read from the compartment volumes, not inferred from sex or the ratio (47).

Shu et al. removed some flow-related heterogeneity by restricting analysis to 448 patients with preserved flow, defined by a left-ventricular ejection fraction of at least 50% and a stroke-volume index greater than 35 mL/m^2^. Calcified volume density correlated with peak velocity and with indexed aortic valve area; associations for noncalcified tissue volume density were weaker and did not rise consistently with severity. Among women with bicuspid valves, severe disease defined by peak velocity was associated with higher noncalcified tissue volume density (*P* = 0.017), but the difference fell short of significance when severity was defined by indexed valve area (*P* = 0.062). In that subgroup the conclusion depended on which severity criterion was applied (48).

These findings do not contradict the associations reported for total volume by Cartlidge and Lembo, because they concern different metrics in different populations. Men made up 78% of the Cartlidge cohort and bicuspid valves 7%; women made up 48.2% of the Shu cohort and bicuspid valves 54.5%; the Lorenzatti cohort had a mean age of 84 years and had already reached TAVR evaluation. Population composition, flow restrictions, and segmentation methods all differed, as did the quantity compared, whether total volume, noncalcified tissue volume, or a ratio between the two. Taken together, these studies show a reasonably consistent association between calcification and stenosis severity, whereas associations with CTA noncalcified tissue vary by population and measurement. Whether noncalcified tissue volume contributes independently to obstruction remains unsettled (36).

### 4.2 Static phenotype, disease activity, and prediction of progression

A single measurement of tissue burden records what has accumulated; local activity and follow-up change describe where the disease is heading. Lembo et al. linked both baseline total volume and its one-year change to gradient progression, which supports total volume as a marker of structural progression. Their imaging analysis drew on SALTIRE2 data, but natural-history association and treatment effect in the parent randomized trial are separate analytical questions. That total volume tracks progression does not mean a component ratio can yet identify patients who will respond to treatment (6).

Lp(a) offers the most coherent chain of evidence from circulating exposure through local activity to progression. In 145 patients, Zheng et al. found greater mineralization activity and faster subsequent progression of CT calcium burden and peak velocity in the high-Lp(a) group, and a biomarker analysis of 220 ASTRONOMER participants associated higher Lp(a) and oxidized phospholipid levels with faster hemodynamic progression (49, 50). A cytokine study of 45 patients with severe stenosis found no clear association between Lp(a) and cross-sectional calcific burden, but existing burden in advanced disease and the rate at which it was acquired are not the same quantity, and a null result in a small cross-sectional sample does not overturn a longitudinal risk association (51).

Baseline distribution also shapes comparisons of progression. Two-year data from 141 patients with mild or moderate stenosis in PROGRESSA showed similar unadjusted change in bicuspid and tricuspid valves; after adjustment for age, baseline severity, and risk factors, bicuspid morphology was associated with faster calcific and hemodynamic progression. Comparisons across morphologic or sex-defined groups depend on starting burden as much as on subsequent rate (52).

### 4.3 Risk stratification and interventional outcomes

Prognostic evidence for CT calcific burden is comparatively mature. In an international registry of 794 patients, greater calcification remained associated with mortality after adjustment for clinical and echocardiographic variables, and a CTA study of 1,521 patients found associations between calcified volume and valve replacement or all-cause death. Mineral burden is, on current evidence, the best established structural marker for risk stratification (40, 53).

Outcome data for noncalcified tissue come mainly from patients already referred for valve replacement. Grodecki et al. compared 447 TAVR patients with 224 controls who had normal valves; tissue composition distinguished certain hemodynamic phenotypes and was associated with short-term adverse events after TAVR. Those events are shaped by ventricular status, anatomy, and the procedure itself, which places them in a different clinical setting from progression of an untreated native valve (54).

Leaflet fibrosis and myocardial fibrosis sit at different points in the disease pathway, and the two are easily conflated. Longitudinal CMR has shown that progressive stenosis is accompanied by myocardial hypertrophy, diffuse interstitial expansion, and accumulating replacement scar; after valve replacement, cellular hypertrophy and part of the diffuse matrix burden regress, while established mid-wall late gadolinium enhancement persists. All of this describes the myocardial response to relief of pressure overload and says nothing about regression of fibrosis in the leaflets themselves (55, 56).

The EVOLVED randomized trial tested a management strategy enriched by a marker of myocardial fibrosis. Among 224 patients with asymptomatic severe stenosis and myocardial fibrosis, the primary composite outcome occurred in 18% assigned to early valve intervention and 23% assigned to conservative management (hazard ratio 0.79; 95% confidence interval [CI], 0.44–1.43), a nonsignificant difference. The trial illustrates the distance between a prognostic marker and a treatment strategy built on it: elevated risk does not guarantee benefit from intervening at a particular moment, and the result applies to the strategy tested rather than to the marker as such (57).

## 5 Pharmacologic intervention and tissue response across disease stages

The studies in this section differ in what they can establish, and Table 3 groups them accordingly. Randomized trials in patients give the most direct estimate of drug efficacy for the population and endpoint studied. Observational medication-exposure studies describe associations shaped by prescribing and baseline differences, and surgical histology describes tissue composition at excision. Animal interventions test mechanisms and treatment responses *in vivo* under defined induction conditions, *ex vivo* intact leaflets resolve local tissue responses, and cell models isolate signaling and cellular activation. Histology–imaging validation (Section 3) addresses measurement accuracy, not therapeutic benefit. When these sources disagree on efficacy, randomized evidence takes precedence.

**Table 3:** Representative intervention and drug-exposure studies, grouped by type of evidence.

Study and intervention	Design and baseline state	Duration	Principal findings
Randomized trials in patients: estimates of treatment effect			
BICATOR/atorvastatin (64)	Randomized trial; 220 patients with bicuspid valves, no severe dysfunction; approximately 56% with a calcium score of zero	3 years	No significant improvement in overall progression of CT calcification or valve dysfunction.
SALTIRE/intensive atorvastatin (65)	Randomized trial; 155 patients with established calcific stenosis	Median 25 months	Progression of CT calcification and peak velocity was not slowed.
ASTRONOMER/rosuvastatin (66)	Randomized trial; 272 patients with mild or moderate stenosis (269 analyzed)	Median 3.5 years	Hemodynamic progression was not improved.
SEAS/simvastatin plus ezetimibe (67)	Randomized trial; 1,873 patients with asymptomatic mild-to-moderate stenosis	Median 52.2 months	Ischemic events were reduced; valve-related events were not.
SALTIRE2/denosumab or alendronic acid (69)	Randomized trial; 152 patients with established calcific stenosis	2 years	Bone-metabolism markers responded as expected, but CT, PET, and echocardiography showed no valvular benefit.
AVADEC/vitamin K2 plus vitamin D (70)	Randomized trial; 389 men with calcium score >300 AU and peak velocity ≤3 m/s	2 years	Calcium score rose by 275 and 292 AU respectively; between-group difference −17 AU (95% CI, −86 to 53).
DIP-CAVD/evogliptin (72)	Randomized trial; 228 patients with heterogeneous baseline calcification	CT 96 weeks; PET 48 weeks	The primary CT volume endpoint did not improve; active calcification volume favored treatment in the PET subgroup.
Ataciguat 2025 (73)	Randomized trial with imaging analysis in 23 patients; moderate calcific stenosis	6 months	CT progression was numerically lower with treatment (*P* = 0.051); change in valve area did not differ significantly (*P* = 0.120).
CaLIPSO/SNF472 (76)	Randomized trial; 274 patients on hemodialysis, 207 in the valvular secondary-endpoint analysis	52 weeks	Valve calcium volume rose by 14% with pooled doses and 98% with placebo; functional benefit for stenosis was not established.
Observational human studies: medication exposure and surgical histology (associations only)			
Carter-Storch 2024/ARB exposure (74)	Observational surgical cohort; 1,321 patients, 89% with severe stenosis	Tissue sampled at surgery	Female ARB users had lower fibrosis scores, with no association in men; change in tissue over time was not measured.
Animal interventions: *in vivo* responses under defined induction conditions			
Miller 2009/2010/genetic lipid lowering plus dietary modification (83, 84)	Reversa mice treated after 6 or 12 months of induction	6 months/2 months	Mineral responses separated from collagen and valve-opening responses; starting stage and exposure differed between experiments.
Chen 2016/AICAR (80)	Klotho-haploinsufficient mice with pre-existing fibrotic remodeling after 13 weeks of high-fat feeding	2 weeks	Terminal collagen burden and RUNX2-related expression fell; evidence was histologic and molecular only.
Bouchareb 2015/2-thioUTP (82)	Mice treated after 8 months of induction, with the same diet continued	60 days	Within-animal micro-CT mineral burden fell from the pretreatment baseline and valve function improved.
*Ex vivo* intact leaflets: local tissue responses			
Feldmann 2023/elastin-targeted DTPA nanoparticles (88)	Intact porcine leaflets treated after 5 days of mineralization	2 days	Terminal tissue calcium content fell; whole-leaflet function under load was not assessed.
Cell models: signaling and cellular activation			
Vogt 2025/optimized drug combinations (89)	Human VICs on hydrogels, treated after 2 or 7 days of activation	2 days	Selected combinations reduced *α*SMA and related activation markers; the endpoint was cellular state.

ARB, angiotensin II receptor blocker; AU, Agatston units; CT, computed tomography; DTPA, diethylenetriaminepentaacetic acid; PET, positron emission tomography; VIC, valvular interstitial cell; *α*SMA, alpha-smooth muscle actin. Groups are ordered by the directness of the evidence they provide on treatment efficacy in patients; animal, *ex vivo*, and cell studies address mechanism and tissue response rather than clinical efficacy. Sample sizes are the number randomized or enrolled unless an endpoint-analysis population is specified; the primary ASTRONOMER analysis included 269 patients. Duration is follow-up for clinical studies and treatment duration for experimental studies.

### 5.1 Starting point of treatment and duration of exposure

Tissue and functional state at the moment treatment begins define the stage at which a drug acts. Detectable mineral, leaflet thickening, functional obstruction, and ongoing mineralization activity do not arrive together, and animal age or induction duration is a poor proxy for any of them. Serial CT and echocardiography give a within-subject baseline; terminal histology usually has to borrow one from a contemporaneous baseline-sacrifice group.

Starting stage and treatment duration are separate variables, and study designs routinely confound them. In a branched-chain amino-acid study, supplementation began at 1, 3, or 6 months of age with assessment in all groups at 12 months, giving exposures of 11, 9, and 6 months. Earlier supplementation reduced leaflet lipid retention without clearly reducing calcium deposition. What the design compared was the cumulative outcome of strategies differing in both starting point and duration, so neither effect can be isolated. Experiments that withdraw a disease-inducing diet when drug treatment starts add a further confounder, combining pharmacologic intervention with a change in etiologic exposure (58).

### 5.2 Lesion formation and early intervention

When treatment starts alongside disease induction, what is being tested is lesion formation. In cholesterol-fed rabbits, atorvastatin reduced terminal leaflet mineralization and Lrp5-related responses. A cross-model study of evogliptin widened the endpoint set to inflammation, fibrosis, and mineralization, and the rabbit arm also showed slower echocardiographic progression. The autotaxin inhibitor BBT-877 similarly attenuated diet-induced fibrocalcific remodeling and functional deterioration. Within their respective induction conditions, these studies support suppression of lesion formation across both tissue and functional endpoints (59–61).

Cell-network and genetic models show that different tissue processes respond differently to the same drug. XCT790 emerged from screening in a human induced-pluripotent-stem-cell–derived model and subsequently reduced valve disease in susceptible mice. In an integrin-linked kinase model, sitagliptin was given from the first day of induced gene deletion: mineralization fell, whereas leaflet thickening and functional abnormality were only partly attenuated. Suppressing an osteogenic response does not necessarily prevent nonmineralized thickening or dysfunction (62, 63).

Randomized evidence in patients has not reproduced these experimental findings. BICATOR randomized 220 patients with bicuspid aortic valves and no severe valvular dysfunction, approximately 56% of whom had a baseline CT calcium score of zero; three years of atorvastatin did not significantly improve overall progression of calcification or valve dysfunction. A calcium score of zero, however, says nothing about noncalcified thickening or congenital valve geometry, and the cohort accordingly mixed incident mineralization with progression of pre-existing burden (64).

Experimental and clinical results diverge for several reasons at once: induction mechanism, target dependence, disease duration, and baseline tissue state. What BICATOR shows is that milder functional disease does not by itself guarantee a valvular response to lipid lowering. The failures of earlier drug trials cannot all be attributed to late treatment initiation, and reading them that way risks repeating the same design with an earlier start and no better result.

### 5.3 Slowing established disease and modifying tissue responses

Lipid-lowering trials show most clearly how allocation shapes what an efficacy result can mean. SALTIRE randomized 155 patients with calcific stenosis; intensive atorvastatin did not slow progression of CT calcification or peak velocity. ASTRONOMER randomized 272 patients with mild or moderate stenosis, 269 of whom entered the primary analysis, and rosuvastatin did not improve hemodynamic progression (65, 66). SEAS, with 1,873 randomized patients, found that simvastatin plus ezetimibe reduced ischemic events but not valve-related events. RAAVE allocated therapy by indication for lipid lowering and observed a slower rise in peak velocity, but its nonrandomized design leaves treatment indication and baseline risk as major confounders. The randomized trials separate vascular benefit from disease modification of the valve (67, 68).

Therapies aimed at mineral regulation have produced negative primary endpoints across the severity range. SALTIRE2 randomized 152 patients; denosumab and alendronic acid produced the expected pharmacodynamic effects on bone metabolism, yet CT, PET, and echocardiography showed no significant valvular benefit. AVADEC enrolled 389 men with a baseline calcium score above 300 AU and peak velocity of 3 m/s or less; after two years the calcium score had risen by 275 AU with vitamin K2 plus vitamin D and by 292 AU with control, a between-group difference of −17 AU (95% CI, −86 to 53). Neither systemic pharmacodynamic effect nor milder hemodynamic disease delivered improvement in the mineral endpoint (69, 70).

An earlier proof-of-concept trial of vitamin K1 had reported slower progression of CT calcification. Later trials differed in formulation, dose, blinding, and baseline population. A favorable signal in a small study and null results in larger randomized trials are evidence of very different weight within the same pathway (71).

DIP-CAVD set a primary mineral-burden endpoint beside an activity endpoint. Among 228 patients randomized to evogliptin or placebo, change in the primary CT calcified-volume endpoint at 96 weeks did not differ significantly, whereas change in active calcification volume favored treatment in the PET subgroup at 48 weeks; tissue-to-background ratio and other measures showed no corresponding effect. The PET and primary CT analyses covered different time points and different participants. The trial provides an exploratory signal for one activity measure; it does not establish a within-patient sequence in which activity falls first and structure improves later (72).

The ataciguat program began from abnormal soluble guanylate cyclase (sGC) signaling in human valve tissue and carried that observation through cellular studies, mice with established disease, and a randomized clinical trial. The six-month clinical imaging analysis included 23 patients. CT calcium score rose from 1,055 to 1,120 AU with treatment and from 1,208 to 1,423 AU in the control group, with a between-group comparison of change yielding *P* = 0.051; for change in valve area, *P* = 0.120. The direction favored slower progression, but sample size and follow-up limited precision. The program is a translational link between tissue mechanism and clinical drug response, not a demonstration of sustained functional benefit (73).

For fibrosis, human evidence comes chiefly from associations between histology and medication exposure. Carter-Storch et al. studied 1,321 patients undergoing surgical valve replacement, 89% of them with severe stenosis, and classified tissue phenotypes by calcification and fibrosis scores in the excised leaflets. Women and patients with bicuspid valves were overrepresented in the fibrosis-dominant group. Among women, use of angiotensin II receptor blockers (ARBs) was associated with a lower fibrosis score (*P* < 0.001); no such association appeared in men, and sex interacted with ARB use. The study links drug exposure to human leaflet tissue, but cross-sectional sampling at the end of the disease course cannot separate pretreatment differences from tissue change under therapy, and prescribing indication and selection for surgery may both have contributed (74).

Randomized evidence on inhibition of the renin–angiotensin system addresses a different structure altogether. RIAS randomized 100 patients with asymptomatic moderate or severe stenosis to ramipril or placebo; at one year, left-ventricular mass changed by −3.9 g and +4.5 g respectively, a significant benefit for the primary endpoint. Change in CMR valve area showed only a trend, and echocardiographic peak velocity did not differ significantly. The ARB histologic association and the RIAS ventricular result are both human evidence about this pathway, but one concerns a fibrotic leaflet phenotype and the other a myocardial response downstream of obstruction. No study has yet measured leaflet composition longitudinally in a way that would join them (75).

Disordered mineral metabolism in dialysis provides a separate treatment context. CaLIPSO randomized 274 patients receiving hemodialysis, with aortic-valve calcification as a secondary endpoint; among the 207 patients in that analysis, calcified volume rose by 14% in the pooled SNF472-dose groups and by 98% with placebo over 52 weeks. In the subgroup with baseline valvular calcification, ADVANCE associated cinacalcet plus low-dose vitamin D with slower percentage progression (76, 77). Valve-calcification endpoints in VitaVasK and Trevasc-HDK were not significantly improved. The favorable findings in this field belong to the dialysis setting and have not been linked consistently to improved stenosis or to fewer valve-related events (78, 79).

Direct pharmacologic responses of established fibrotic disease are reported more often in experimental work. Klotho-haploinsufficient mice given the AMP-activated protein kinase agonist AICAR for two weeks after 13 weeks of a high-fat diet showed lower terminal collagen burden and RUNX2-related expression. A protease-activated receptor 2–targeted nanocarrier given after a longer period of high-fat feeding reduced terminal fibrosis, mineralization, and leaflet thickening, accompanied by lower peak velocity. Ongoing lesions can evidently remain responsive, though the tissue evidence rests on terminal between-group comparison rather than on change measured within an animal (80, 81).

### 5.4 Structural regression and functional recovery

Bouchareb et al. provide longitudinal evidence of mineral regression in mice. The animals were kept on a high-fat, high-carbohydrate diet for eight months and had elevated peak velocity before receiving 2-thioUTP, with the disease-inducing diet continued throughout. Micro-CT in the same animals at baseline and after 60 days showed reduced mineral burden together with improved valve function. Unlike studies confined to terminal tissue differences, this design tracked change in both pre-existing mineral and function (82).

The Reversa model separated tissue endpoints from one another. After six months of induction, genetic lipid lowering combined with dietary modification prevented further functional deterioration and pushed mineral burden below that of a contemporaneous baseline group, while collagen did not fall. After 12 months of induction, a similar intervention altered mineral and osteogenic measures without restoring valve opening. Treatment lasted six months in the first experiment and two in the second, so stage and exposure both differed. What held across both was the capacity of mineralization responses to separate from changes in collagen and in valve opening (83, 84).

Apolipoprotein A-I and mimetic therapies have mostly produced short-term functional signals. In rabbits, Busseuil et al. observed an increase in valve area with a group-by-time interaction. Speidl et al. reported improvement in valve area and peak velocity on day 8 after two ETC-216 infusions, resting largely on paired within-group comparison. Both experiments simultaneously attenuated the dietary stimulus, and mineral was measured only at terminal sampling, so no within-subject correspondence between functional change and net mineral reduction is available (85, 86). In mice kept on a high-fat diet, valve area changed favorably while peak velocity continued to rise in both groups and terminal mineralization of the aortic sinus did not differ clearly. Discordance across anatomic regions and functional measures makes it impossible to attribute the valve-area change to mineral regression alone (87).

*Ex vivo* leaflets and cell cultures each capture a single response. Feldmann et al. first mineralized intact porcine leaflets, then applied elastin-targeted nanoparticles carrying diethylenetriaminepentaacetic acid (DTPA); terminal tissue calcium content fell and cell viability was assessed, but leaflet opening and closing under load were not measured. In human VICs, Vogt et al. applied sex-optimized drug combinations after activation, and selected combinations reduced *α*SMA and related markers. Neither system tests whether lower tissue calcium or reduced cellular activation restores the collagen network or valve function (88, 89).

The experimental literature establishes that some mineral lesions and cell states remain modifiable, with functional improvement observed in a small number of models. Human pharmacologic studies still center on progression endpoints, and longitudinal measurement of the leaflet fibrous matrix is almost absent. That gap limits how far structural and functional discrepancies can be interpreted, but it is not, on present evidence, a sufficient explanation for the run of negative trials.

Table 3 summarizes representative intervention and human drug-exposure studies.

## 6 Discussion: therapeutic objectives and multilevel efficacy assessment

### 6.1 Defining prevention, slowing, and reversal

Prevention, slowing, and reversal rest on different baselines and different comparisons. Prevention starts in participants without detectable target pathology and compares the incidence of new lesions or time to onset. Slowing starts from established pathology and compares baseline-adjusted increments or rates of progression. Reversal requires a fall in lesion burden from the pretreatment baseline, with the treatment effect estimated from the between-group difference in change. A terminal burden that lies above baseline but below the control group is slower progression, not reversal, a distinction frequently blurred in the way results are described. In cohorts containing both zero and positive baseline calcium scores, incident calcification and progression of existing calcification have to be estimated separately. A fall in tissue burden is a structural outcome and improvement in valve function a functional one; their magnitudes and time courses need not agree.

Pharmacodynamic measures, tissue change, function, and clinical events sit at different levels of the same question. The contrast between ischemic and valve-related events in SEAS, and between ventricular mass and leaflet obstruction in RIAS, shows why the anatomic and biological location of a benefit matters. Valve replacement, heart-failure hospitalization, and death have different determinants, and the components of a composite endpoint reta\n those differences even when the composite does not. The prognostic association of an imaging measure makes it a candidate endpoint; whether treatment-induced change in that measure predicts clinical benefit is a separate claim requiring separate validation.

### 6.2 Prognostic and treatment-response markers

A faster-progressing phenotype improves the detectability of structural change over a given follow-up, which makes it useful for risk enrichment. A phenotype that predicts response to a particular therapy is a different proposition and requires a demonstrated interaction between treatment and the baseline marker. Continuous measures of calcified burden, noncalcified burden, and activity preserve the information gradient; a single cut point that defines a phenotype by relative noncalcified or calcified volume is hostage to the distribution of the cohort in which it was derived.

Patient-derived material offers experimental evidence of response heterogeneity. Vogt et al. combined serum proteomics with VIC drug screening and found that serum insulin-like growth factor-binding protein 2 (IGFBP2) in women with aortic stenosis was associated with the capacity of evogliptin to suppress myofibroblast activation; neutralizing IGFBP2 or inhibiting Rho/ROCK and focal-adhesion-kinase signaling restored part of the response. The finding links the serum environment to cellular drug sensitivity, measured in culture rather than in valve tissue (90).

Neither observation establishes sex as a basis for treatment selection. The ARB finding is an observational association in excised tissue, and the serum-based platform yields an experimental signal in cultured cells. Both justify testing sex as an effect modifier in studies that measure tissue and microenvironmental features directly; neither allows sex to stand in for an individual's tissue composition or to serve as a validated selection marker for antifibrotic therapy.

### 6.3 Longitudinal measurement and interpretation of discordant endpoints

Detecting slow progression or modest regression is first a question of repeatability. Repeat-CT studies have shown that standardized calcium scoring tracks progression of aortic-valve disease with high sensitivity and, once repeatability is accounted for, outperformed the echocardiographic measures assessed in that study. Group-level drug effects are judged from between-group change and confidence intervals; credible change in an individual patient is judged against measurement error. The two use different standards and should not be reported interchangeably (91).

Discordant endpoints have both biological and technical origins. Activity, mineral retention, and tissue remodeling may follow different time courses, and flow conditions can move echocardiographic measures independently of the leaflet. Differences in participant subsets, follow-up intervals, segmentation boundaries, and repeatability will also drive results apart. The PET and CT findings in the randomized DIP-CAVD trial, and the divergence between valve area and peak velocity in short-term animal apolipoprotein A-I experiments, illustrate the problem. Discordance alone identifies no mechanism; longitudinal multimodality data from the same individuals are what make interpretation possible.

Statistical unit and missing-data mechanism deserve more attention than they usually receive. The animal, donor, or patient is the independent unit, whereas leaflets, sections, and microscopic fields are nested measurements. Patients who progress fastest may undergo valve replacement earliest and so contribute no final native-valve scan, which means that analyses restricted to those completing imaging follow-up can underestimate structural progression.

Several analytic strategies can limit this bias. Joint longitudinal–time-to-event models link serial CT or CTA trajectories with time to valve replacement and death, treating the two as distinct events that end native-valve follow-up (92). Inverse-probability-of-censoring weighting is an alternative when dropout is explained by recorded clinical and imaging history and observation probabilities remain positive (93). Both depend on adequately specified models, and prespecified tipping-point analyses show how conclusions change under different assumptions about unobserved values. The estimand should also be explicit: progression before replacement differs from a hypothetical trajectory without replacement, which requires model-based extrapolation because post-replacement imaging no longer reflects the native valve (94). Replacement and death are then analyzed with the structural trajectory rather than treated as missed scans.

### 6.4 Phenotype stratification and directions for interventional research

Histologic validation of CTA has so far involved mainly advanced valves. Evidence on component identification and repeatability is thin in milder disease and across sex and valve-morphology strata. Three questions stand between imaging characterization and therapeutic application: what tissue actually corresponds to early noncalcified thickening, what noncalcified burden adds beyond calcification, and whether pretreatment phenotype modifies drug effect. Comparisons across disease stages also require equal treatment duration, without which the effect of starting earlier cannot be told apart from the effect of treating longer.

New pharmacologic protocols have adopted multimodality endpoints. CHIANTI plans to enroll 150 patients with moderate stenosis and compare low-dose colchicine with placebo, with the 24-month CT calcium score as the primary endpoint and PET uptake and peak velocity as secondary endpoints. PASSPORT tests vitamin K1 in mild or moderate calcific stenosis, combining CT and echocardiography to assess progression. Both link activity, mineral burden, and function; neither has yet reported efficacy results (95, 96).

Dynamic risk tools offer one route into clinical practice. A web-based calculator could combine serial echocardiography, separately quantified CTA calcified and noncalcified tissue volumes, and selected circulating biomarkers, updating the risk of a prespecified progression or clinical-event endpoint as new measurements arrive. The ASteRisk score, an online calculator of clinical and echocardiographic risk in aortic stenosis, provides a precedent (97). The interface could show the prediction horizon, uncertainty, and missing inputs at each visit. Omics-derived signatures would enter as candidate predictors and be retained only if they add information beyond routine clinical and echocardiographic data.

For complex models, SHapley Additive exPlanations (SHAP) can show each input's contribution to an individual prediction; these values are model attributions, not biological causes or treatment effects (98). Observed trajectories could be displayed beside the updated risk, with discordant measurements flagged for clinical review. External validation and calibration across sites, sex, and valve morphology, followed by prospective evaluation of workflow effects and clinical utility, would precede routine use (99). The first applications would be follow-up planning and risk enrichment for interventional studies.

This synthesis has limitations. Purposive selection, screening without duplicate review, and the absence of formal risk-of-bias assessment leave it open to selection bias, and citation-ranked retrieval of database records favors established, highly cited studies over recent or less visible work. English-language sources predominate. Experimental work is reported selectively, and heterogeneity in species, induction protocol, and endpoint definition precludes quantitative pooling. Several of the imaging studies cited here also share investigators, cohorts, or analysis software, so agreement among them should not be read as full independence.

Links between medication exposure and human histology have begun to appear, and *in vivo* longitudinal measurement of tissue composition now has an initial evidence base. What is still missing is the bridge between them: baseline tissue phenotype, treatment-associated change in composition within the same patient, and the relationship of that change to function and to clinical events. Until studies are designed to supply that chain, the role of fibrocalcific phenotype in treatment stratification and efficacy assessment will remain a hypothesis rather than a tool.

## 7 Conclusions

Fibrosis and calcification together alter the structure and mechanics of the aortic valve. Tissue composition varies between individuals and shows group-level associations with sex, age, and valve morphology. Calcific burden has consistent diagnostic and prognostic value. CTA-derived total fibrocalcific volume has been validated against tissue mass and has preliminary longitudinal support, whereas the independent clinical value of CTA noncalcified tissue volume remains dependent on method and population.

Treatment studies measure mineral progression far more often than they measure the response of the leaflet fibrous matrix. Observational human data associate ARB exposure with leaflet tissue phenotype, and animal and cell studies show that mineral, cellular, and functional responses can dissociate, but there is no randomized evidence for phenotype-guided drug selection. Progress will depend on defining the lesion state at baseline, separating incidence from progression, confirming change in pre-existing burden, and demonstrating that a tissue response translates into durable functional benefit. Trials that measure leaflet composition longitudinally, in the same patients, are the most direct route to that evidence. Once validated, dynamic risk tools could carry these measurements into individualized follow-up.
